# Problems of HLAW Hybrid Welding of S1300QL Steel

**DOI:** 10.3390/ma15165756

**Published:** 2022-08-20

**Authors:** Bogdan Kupiec, Michał Urbańczyk, Magdalena Radoń, Marek Mróz

**Affiliations:** 1 The Department of Casting and Welding, Faculty of Mechanical Engineering and Aeronautics, Rzeszow University of Technology, Al. Powstancow Warszawy 12, 35-959 Rzeszow, Poland; 2 Lukasiewicz Resarch Network—Institute of Welding, Błogoslawionego Czeslawa 16-18, 44-100 Gliwice, Poland

**Keywords:** hybrid welding, S1300QL steel, microstructure, mechanical properties

## Abstract

This paper presents the results of tests on the fabrication of welded joints in S1300QL steel according to the requirements of ISO 15614-14 and ISO 12932. The butt-welded joint without bevel was made from 350 × 150 × 8 mm sheets. The welding process was carried out at the hybrid welding (laser–MAG) station. MAG means metal active gas. The test welded joints were subjected to non-destructive and destructive testing. Visual and radiographic examinations were carried out. The distribution of HV10 hardness was determined in the weld, the heat-affected zone, and the base material. The microstructure of these areas was also analysed for the presence of hard and brittle hardening products and non-metallic inclusions. Tensile strength and yield strength, as well as bending strength, were assessed in the mechanical property tests. The impact test was performed in accordance with ISO 9016.

## 1. Introduction

Increasing demands on welded structures for high durability and reliability, as well as operational safety, are forcing the use of modern construction materials with improved strength parameters.

Modern structural materials should be characterised by high yield strength, good ductility and low brittleness, good weldability, and good impact strength, while maintaining a relatively low price. These materials can be used for heavily loaded machine parts and welded structures, such as cranes, gantries, bridges, tanks, mining machinery, and equipment [1,2,3].

Modern structural materials include structural steel with improved yield strength in the tempered state, classified in EN 10025-6 [4]. In the cases of S960QL and S1100QL steel, which haves minimum yield strengths of 960 and 1100 MPa, respectively, the fine-grained microstructures consist of martensite or martensite and bainite, i.e., the microstructural components obtained as a result of the tempering treatment.

Further development of the material group of higher-yield-strength steel has led to the development of ultra-high-strength materials with yield strengths of up to 1300 MPa, for example steel S1300QL [5], and even up to 1700 MPa [6]. These steels belong to the group of UHSS steels (ultra-high-strength steels) [7]. The Swedish company SSAB, which produces HARDOX, WELDOX, ARMOX, or TOOLOX steel, as well as TyssenKrupp [8,9,10,11], have become pioneers among the world’s producers of this type of material.

The occurrence of hard and brittle structural components (hardening products) in high-yield-strength steel is the reason for the limited weldability of these materials, especially in terms of microstructural changes occurring in the heat-affected zone (HAZ) [12,13]. During the welding process, the action of the heat source results not only in the formation of a pool of liquid metal with subsequent solidification of the weld area, but also in changes to the microstructure of the HAZ as a result of phase transformations in the solid state. The austenitisation of the base material, which takes place during the welding process, results in the growth of austenite grains and the occurrence of under-hardened areas, which can lead to the nucleation and propagation of cracks. In the dimension of mechanical properties, this results in a reduction in ductility and impact strength, as shown in the work [14,15].

Research into the development of welding technology for higher-yield-strength steel seeks to minimise the HAZ by modelling the welding process through the selection of appropriate process parameters. The tangible result of these measures should be a welded joint with high plastic properties. For example, the authors of [16] show that the use of a gas mixture of 98% Ar + O_2_ results in a welded joint of S690QL and S960MC steel sheets with a yield strength of 435 MPa (R_e_) and good strength properties (R_m_) of 717 MPa.

**Figure 1:**
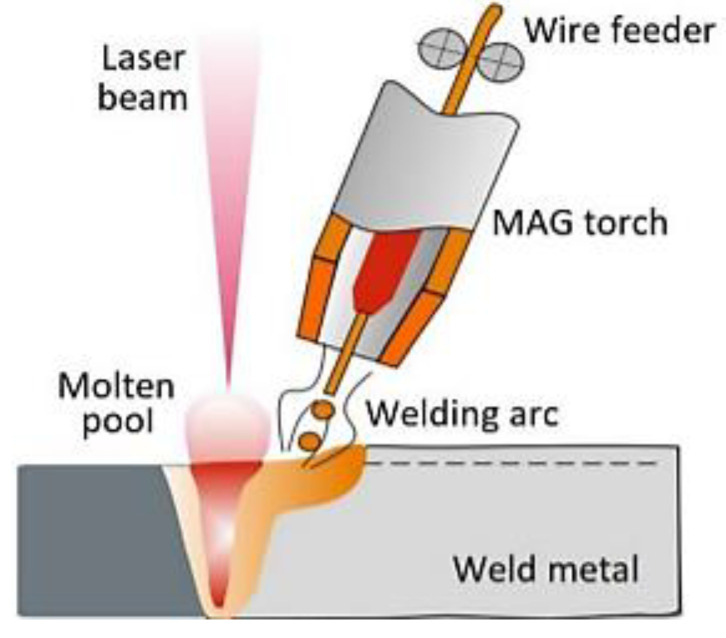
Schematic diagram of HLAW [19].

Recently, hybrid laser-arc welding (HLAW) has attracted considerable interest [17,18,19]. A schematic diagram of the HLAW method is shown in Figure 1 [20].

Urbańczyk M. et al. [20] analysed the mechanical properties and microstructure of a welded joint made of S960QL steel using the HLAW method. Turichin G. et al. [21] analysed the effect of the chemical composition of the binder on the microstructure and mechanical properties of welded joints of ARMOX, WELDOX, and HARDOX steel fabricated using the HLAW method. Mazar Atabaki M. et al. [22] investigated the feasibility of using HLAW to join advanced high-strength steel (AHSS) components with yield strength Re = 1034 MPa. The authors of the paper [23] indicate that the HLAW method can be successfully used to produce welded joints in S960QL steel in terms of meeting the requirements of EN ISO 15614-14. The author of the paper [24] presents the possibility of fabricating welded joints from steel grades of different thickness values by hybrid welding using a 32 kW laser.

It is now becoming a major challenge to develop correct welding technology for steel with a yield strength exceeding 1300 MPa (steel grade S1300QL). Many authors have tried to apply methods such as HPAW (hybrid plasma-arc welding) [25], TIG, A-TiG and MAG [26], or Laser [10,11] to weld parts made of this steel.

The aim of the study was to assess the feasibility of welded joints in S1300QL steel using the hybrid welding method (HLAW) in terms of meeting the requirements of ISO 15614-14 and ISO 12932.

## 2. Materials and Methods

In this study, 8 mm-thick sheets of high-strength tempered S1300QL steel were selected for testing. The chemical composition and mechanical properties according to EN 10025-6, the manufacturer’s certificate, and that determined by control chemical analysis are given in Table 1. The control analysis of the chemical composition of the steel was carried out using Bruker Q4 TASMAN spark spectrometer (AXS, Kalkar, Germany).

The supplementary material used during welding was a wire with the trade name BÖHLER Union X96 with a diameter of 1.2 mm (Vienna, Austria). This welding wire made a low-alloyed solid wire of high-strength, fine-grained steel. The chemical composition of the wire according to the certificate and control analysis is shown in Table 2.

Based on the chemical inventory of the EN test, it is equivalent to CEV ISO 1011-2 Formula (1). This pattern is also provided by the MIS (International Welding Institute, Paris, France).

(1) $$\mathrm{CEV}=C+\frac{\mathrm{Mn}}{6}+\frac{\mathrm{Cr}+\mathrm{Mo}+V}{5}+\;\frac{\mathrm{Ni}+\mathrm{Cu}}{15}\%$$

From the point of view of carbon content, carbon steels can be divided into: well weldable (maximum 0.25% C), sufficiently weldable (0.25–0.35% C), with limited weldability (0.35–0.45% C), and badly weldable (>0.45% C). The calculated equivalent was ≈ 0.44%, which means that this steel is characterised by limited weldability, which was also demonstrated by the authors of research [27,28,29,30].

The test joints were made from sheets of S1300QL steel measuring 350 × 150 × 8 mm. The sheets were not bevelled (“I” butt joint, no clearance/gap). Once the sheets were cleaned, tack welds were made, and the welding process began. The welding process was carried out after preheating to 200 °C. According to [24], this temperature is defined as the maximum interpass temperature when welding S1300QL steel.

**Figure 2:**
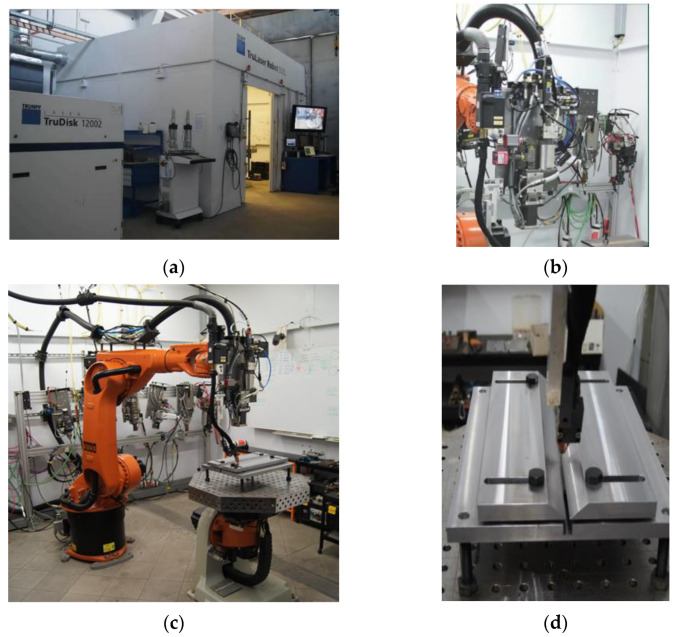
Robotic laser welding station (TruLaser Robot 5120) with a TruDisk 12,002 disk laser and a D70 hybrid welding head (Trumpf): (**a**) view of the booth and laser source, (**b**) hybrid head, (**c**) view of the robot with welding table, (**d**) how the welding sample is fixed.

The welding process was carried out using a robotised Laser–MAG hybrid welding station, which is owned by the Łukasiewicz Research Network—Welding Institute (Figure 2). The station consists of a 12 kW TruDisk 12,002 disk laser (TRUMPF, Stuttgart, Germany), a KRC30HA welding robot (KUKA, Augsburg, Germany) equipped with a hybrid welding head and a PHOENIX 452 RC PULS MIG/MAG welding machine (Shanghai, China), which allows welding with a maximum current of 450A (EWM Hightec Welding GmbH, Mündersbach, Germany).

**Figure 3:**
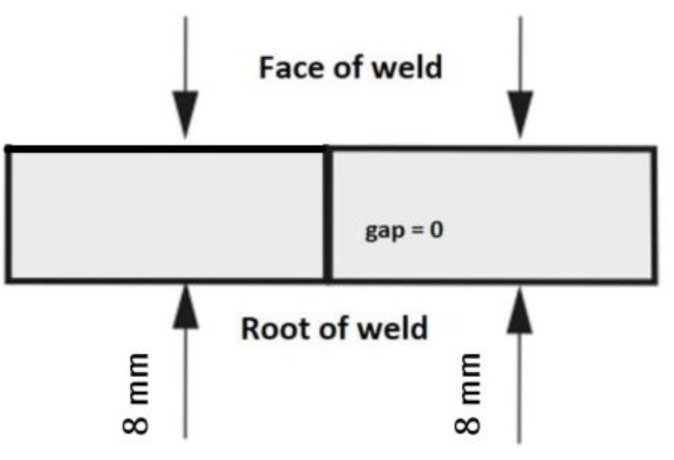
Position of the plates in the flat position (PA) according to the positions of the ISO standard.

The welding process was carried out in the flat position (PA) (Figure 3). The welding process parameters are shown in Table 3.

The S1300QL steel welded joints were subjected to non-destructive and destructive testing.

Non-destructive testing is visual and radiographic. Visual testing was carried out in accordance with the requirements of ISO 17637. The macroscopic metallographic examination was performed using a Zeiss Neophot 2 microscope (Zeiss, Jena, Germany). To determine the macrostructure, the specimens were etched with Adler’s reagent (Chmes, Poznań, Poland). Radiographic testing of the welded joints was carried out in accordance with the requirements of ISO 17636-1. An Eresco 65 MF3 X-ray machine (GE Sensing&Inspection Technologies; Ahrensburg, Germany) was used.

Metallographic tests to analyse the microstructure were carried out in accordance with the requirements of ISO 17639 on metallographic specimens taken from sample welded joints. In the preparation of the metallographic specimens, the samples were ground with 800- and 1000-grit sandpaper, polished using an ATM Saphir 320E polisher with an ATM Rubin 500 head (Mammelzen, Germany) and Struers’ M-type monocrystalline diamond suspension (3 µm), and etched in 5% Nital (5% HNO3 in ethanol). The metallographic specimens were observed using a Zeiss Neophot 2 light microscope (Zeiss Jena, Germany) and a scanning electron microscope (SEM- VEGA 3 TESCAN, Brno, Czech Republic).

Hardness distribution tests were carried out in accordance with the requirements of ISO 9015-1 using a GNEHM DIGITAL BRICKERS 220 hardness tester. Vickers hardness (HV10) measurements were carried out along two measuring lines 2 mm from the top and bottom edges of the specimen. Impressions were made in the base material, the heat-affected zone (HAZ), and the weld.

A static tensile test was carried out in accordance with the requirements of ISO 6892-1 using 300 mm × 25 mm × 5.0 mm specimens. Excessive root and face height was rectified during the sample preparation process. The tests were carried out on an MTS 810 TEST SYSTEMS testing machine (Eden Prairie, MN, USA).

The weld-face tensile bend test (FBB) and weld-root tensile test (RBB) were performed according to the requirements of ISO 5173. The tests included four specimens—two specimens on each side. The tests were conducted on specimens measuring 300 mm × 20 mm × 5.0 mm. The tests were carried out on an LOS12126 testing machine (Losenhausenwerk AG; Düsseldorf, Germany).

Impact tests were performed in accordance with ISO 9016. Two sets of specimens were used in the tests. The first set was taken from the weld area and the second set from the heat-affected zone. There were three specimens in each set. The thickness of the test joint sheets made it impossible to take impact test specimens of standard dimensions (10 × 10 × 55mm), so specimens with reduced cross sections (2.5 × 8.0 × 55mm) were taken. V-notched specimens were used with a notch depth of 2 mm. The specimens were cooled to −40 °C before testing. The cooling process was carried out using an FP89 cooling circulator (Julabo, Stamford, UK). Impact tests were carried out using an RKP 300 impact machine (Amsler, Colombo, Sri Lanka).

## 3. Results and Discussion

### 3.1. Visual and Macroscopic Examination

Visual examination showed that the welded joint was characterised by a smooth face without splatter and a correctly executed root. Non-conformity in No. 502 was observed, i.e., there was excess weld metal according to EN ISO 6520-1. The height of the weld face was 2.3 mm. The completed welded joint based on ISO 12932 met the relevant criteria and represented quality level B, while the excess weld metal met the requirements of quality level D.

**Figure 4:**
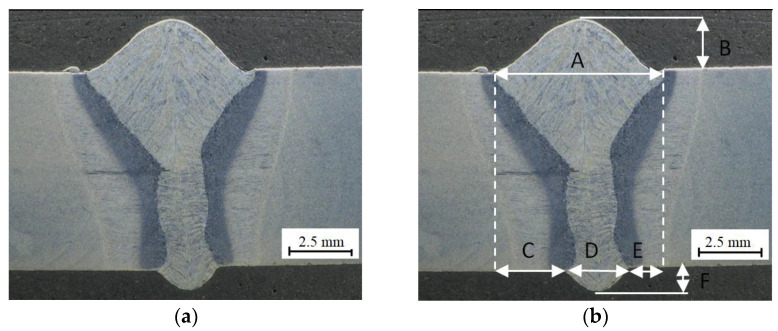
Joint in S1300QL steel: (**a**) macrostructure, (**b**) weld dimensions: A—width of the weld face, D—width of the weld root, C, E—shift of the weld root relative to the weld face, F—height of the weld root.

The macrostructure and dimensions of the welded joint in S1300QL steel are shown in Figure 4.

The metallographic macroscopic examination revealed misalignment of the weld. A slight offset was found at the top of the weld (weld face), visible in Figure 4b, which may be due to non-axial feeding of the electrode wire during welding. The width of the weld face (A) of the joint made in the PA position was 7 mm, while its face height (B) was 2.3 mm. The width of the weld root (D) is 2.5 mm, and its height (F) is 0.8 mm. A photo of the macrostructure revealed a dendritic weld and a clear heat-affected zone. No cracks were observed in this joint.

After visual and macroscopic examinations, the analysed welded joint was found to comply with quality level B, while the excess weld metal met the requirements of quality level D according to the requirements of ISO 12932 for the qualification of the hybrid welding procedure.

### 3.2. Radiographic Examinations

**Figure 5:**
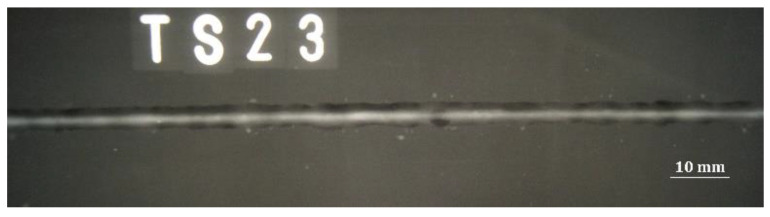
Photo of a test of a hybrid welded joint in S1300QL steel.

Examples of radiographic results are shown in Figure 5. Analysis of the radiographs, taken along the entire length of the welded joint, makes it possible to conclude that there are no internal defects in the area.

### 3.3. Hardness Distribution

**Figure 6:**
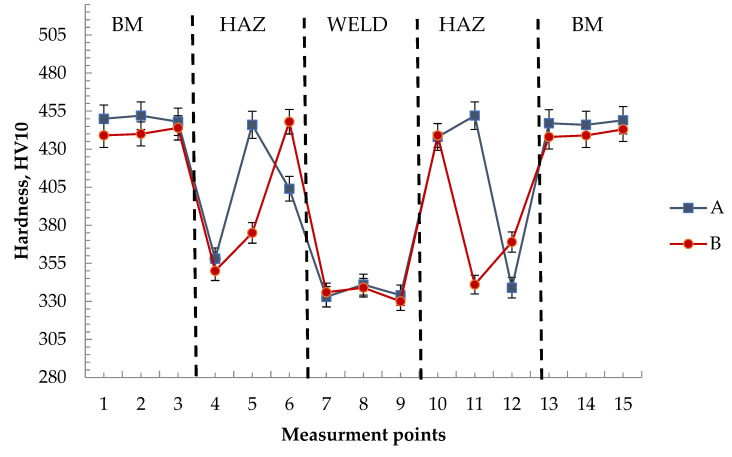
Hardness distribution in the tested welded joint—average result.

The results of the hardness distribution in the different zones of the welded joint are shown in Figure 6. A maximum hardness of 458 HV10 was observed in the HAZ (point 11 of measuring line A). The hardness in the weld axis was 344 HV10. The hardness at the base material–HAZ transition line (point 12 of measuring line A) was 373 HV10. The hardness of the base material ranged from 436 ÷ 455 HV10. The average results of the hardness test are presented in Table 4.

According to the requirements of PN-EN ISO 15614-14 [31], the maximum permissible joint hardness for the material group according to ISO/TR 15608 [32] is 450 HV10, with special values to be agreed with the customer for Re > 890 MPa steel.

When qualifying the welding technology of S1300Ql steel, it is proposed to take the maximum hardness of the base material as a criterion for the allowable hardness value of this joint. The maximum HV10 hardness of the base material is 452 HV10. By analysing the hardness distributions in the weld and in the heat-affected zone, it was found that this criterion is fulfilled.

### 3.4. Welded Joint Microstructure

**Figure 7:**
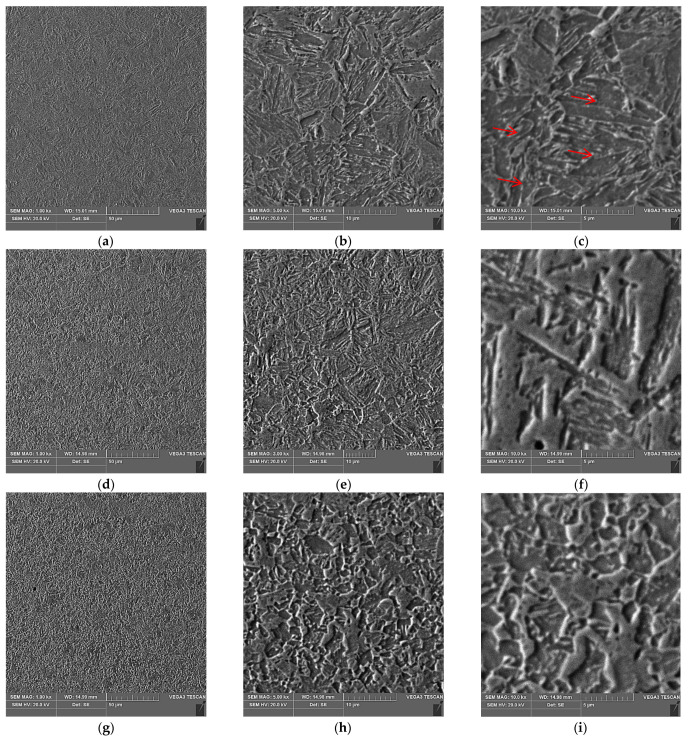
Microstructure of the welded joint in the following characteristic areas: (**a**–**c**) base material; (**d**–**f**) weld; (**g**–**i**) HAZ. The red arrows indicate an example dispersive carbide and carbide–nitride precipitates.

Figure 7 shows the microstructure of the base material (BM), the HAZ, and the weld joint of S1300QL steel.

The base material is characterised by a fine-grained martensitic and martensitic–bainitic structure, with dispersive carbide and carbide–nitride precipitates, as shown in Figure 7c, occurring at the borders of the martensite strips and in their interior. Bainite mostly occurs within grain boundaries. Similar conclusions were reached by the authors [2].

The HAZ area is characterised by a microstructure with fine martensite precipitates and an area heated in the Ac1 ÷ Ac3 range (partial recrystallization area), in which bright martensite fields are observed against the dark etching structure of highly moderated martensite. 

A martensitic structure is observed throughout the weld. From the middle of the weld to the face, the columnar crystals are arranged from the fusion line to the face, as shown in Figure 4a. This is due to the large width of the joint and slower cooling compared to the other half of the joint. On the other hand, the lower half of the weld (to the root), in Figure 4a, is characterised by columnar crystals arranged from the fusion line to the weld axis. This is due to the small width of the joint and the rapid dissipation of heat to the base material. Due to the high purity of the steel (low S and P content), no impurity band is observed in the area where the crystals meet.

### 3.5. Static Tensile Test

The results of the static tensile test of the samples taken from the S1300QL steel joint are shown in Table 5. The basis for accepting the conformity of a welded joint is the achievement of the required minimum tensile strength of the base material, Rm ≥ 1400 MPa. In the case of test joints R1 and R2 analysed, tensile strength values of 171 and 114 MPa less were obtained, respectively, which is the basis for concluding that the analysed joint does not comply with the tensile strength requirements.

**Figure 8:**
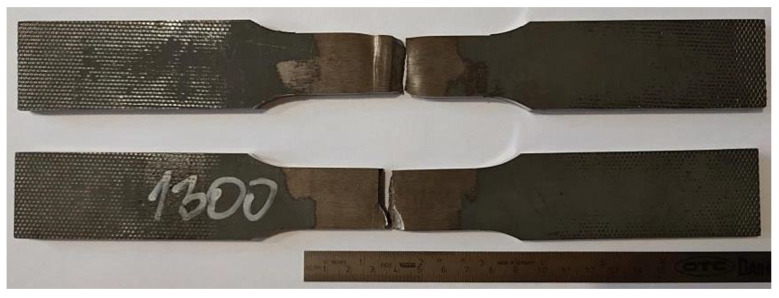
View of the specimens after tensile testing.

A view of the specimens after tensile testing is shown in Figure 8. Rupture of the specimens occurred in the heat-affected zone.

**Figure 9:**
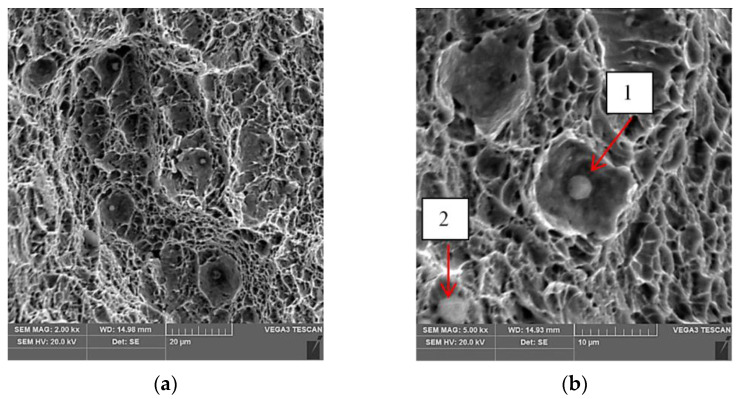
SEM image of the fracture surface of specimen R1 after tensile testing: (**a**) magnification 2.00 kx; (**b**) points 1 and 2 mark the places where a quantitative point X-ray microanalysis was performed, magnification 5.00 kx.

The observational results of the fracture surfaces of the specimens after the tensile tests are shown in Figure 9.

The fracture surface was found to have the characteristic features found in ductile fracture. On the surface of the fracture, there are areas with spherical particles of intermetallic phases Figure 9a,b. Similar precipitates in the fracture were also found by the authors of the paper [1], who analysed the fractures of welded joint specimens in Weldox 1100 steel. For phase identification, spot quantitative X-ray microanalysis was performed according to the designation given for Figure 9b. The results of this analysis are shown in Table 6.

The presence of calcium and aluminium in the spherical particles of the intermetallic phase, according to the results of [1], suggests that the spherical particles are calcium aluminate (CaO-Al2O3). These particles have a detrimental effect on the fatigue strength of the material.

### 3.6. Bending Test

The results of the bending test on the S1300QL steel joints are shown in Table 7.

**Figure 10:**
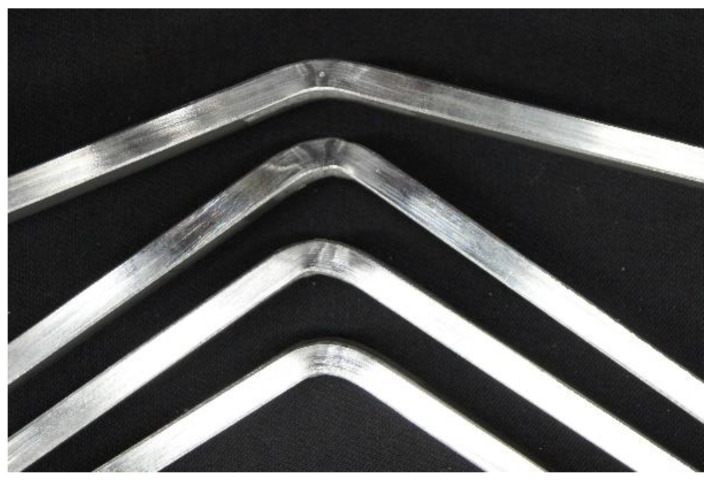
View of specimens after bending.

According to the criterion adopted, the value of the maximum bending angle should be 90° in the absence of visible signs of specimen fracture. In the case of three specimens, the angle value exceeded 60°. The bending angle for the FBB/II and RBB/II samples was 70°, while for the RBB/I sample it was 65°. Observation of the specimen surface after bending revealed the presence of brittle cracks in the heat-affected zone. If an angle value of less than 90° is achieved and brittle cracks are present, the basic criterion for the bending test is not met. Figure 10 shows all 4 samples after the bending test.

### 3.7. Impact Testing

The results of the impact tests at −40 °C are shown in Table 8.

**Figure 11:**
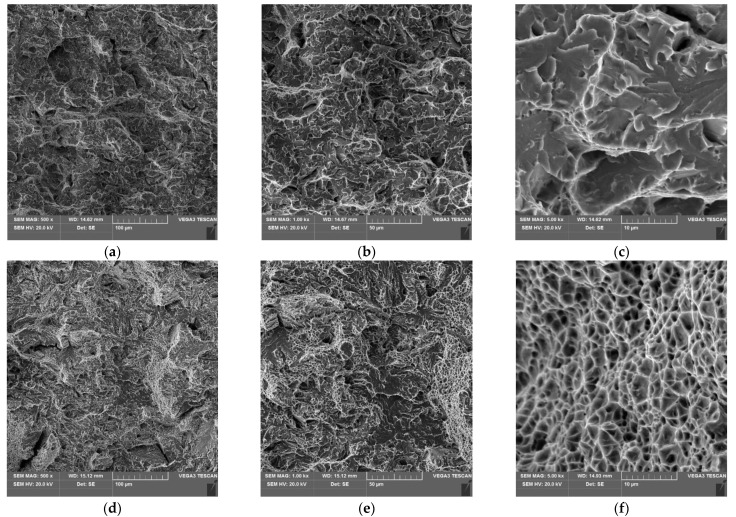
SEM image of the fracture surface of the specimens after impact testing; (**a**–**c**) VWT specimens, (**d**–**f**) VHT specimens.

The results show that the criterion that the breaking work should be ≥18 J was only met for specimens 4, 5, and 6 where the impact strength of the heat-affected zone was assessed. For the specimens where the notch was in the weld area material, the breaking work only for specimen 2 was within the acceptable range. It must therefore be concluded that the impact test results are not satisfactory and do not meet the requirements of ISO 15614-14. This is also confirmed by observations of the fractures of the specimens, the results of which are shown in Figure 11.

On the fracture surface of the specimens with a notch in the weld (VWT specimens), the interaction between brittle and ductile cracking mechanisms (mixed fracture) was found, with a dominant effect of brittle cracking.

For notched specimens in the HAZ, ductile cracking is the dominant mechanism for propagation of the main crack.

## 4. Conclusions

This paper presents the results of qualification studies on butt-welded joints made using a hybrid method (laser + MAG) made of S1300QL grade steel sheets.

- Qualification of the test joints was performed based on non-destructive testing and destructive testing.
- The welded joints were characterised by an even, smooth face with no visible spalling. The root was characterised by its correct formation.
- The visual and radiographic examination did not reveal the presence of surface defects.
- The metallographic examination showed that the weld is characterised by a martensitic structure. Columnar crystals are arranged from the fusion line to the weld axis. The heat-affected zone (HAZ) contains minor precipitates of martensite.
- The static tensile test showed that the produced joints were characterised by high strength but did not meet the accepted requirements. These joints failed to meet the minimum tensile strength requirements of the base material, i.e., Rm ≥ 1400 MPa.
- A maximum bending angle of 70° was found in the bending test, which did not meet the requirements stipulating that for S1300QL steel the required bending angle should be 90°.
- The impact tests showed that the condition for the required minimum value of breaking work was met for notched specimens in the HAZ. In the case of the specimens with a notch cut into the weld, the minimum breaking work was not achieved, which was the basis for the unsatisfactory impact test results.
- The analysis of the test results shows that obtaining a high-quality joint using S1300QL steel that meets the requirements of the standards is difficult and requires further research. The problem of achieving a proper welded joint is the formation of hard and brittle hardening products during welding, as well as the presence of spherical calcium aluminate particles (CaO-Al_2_O_3_), which have a detrimental effect on the strength properties of the material.

## Figures and Tables

**Table 1:** Chemical composition and mechanical properties of steel grade S1300QL.

Chemical Composition, (%)										
	C	Si	Mn	Cr	Ti	Ni	Mo	Nb	V	Cu
Standard	0.25	0.50	1.40	0.80	0.02	2.0	0.70	0.04	0.08	0.10
Certificate	0.24	0.22	0.89	0.49	0.004	1.3	0.392	0.021	0.02	0.01
Control test	0.20	0.212	0.87	0.50	0.002	1.23	0.391	0.021	0.019	0.01
Mechanical properties										
R_m_ [MPa] 1400–1700			R_e_ [MPa] min. 1300				A_5_ [%] min. 8			

**Table 2:** Chemical composition of the Union X96 welding wire.

	C	Si	Mn	Cr	Mo	Ni
Certificate	0.10	0.80	1.80	0.35	0.60	2.30
Control test	0.11	0.78	1.93	0.43	0.55	2.31

**Table 3:** Parameters of hybrid welding of S1300QL steel.

P, kW	V_s_, m/min	V_d_, m/min	I, A	U, V	kor. U, V	Q, kJ/mm	T_p_, °C
4.5	1.2	8.5	275	27	3	0.59	200

P—laser power, V_s_—welding speed, V_d_—wire feed speed, I—current, U—voltage, cor. U, V—correction, Q—heat input, T_p_—sheet pre-heating temperature.

**Table 4:** Average hardness test results.

	BM			HAZ			WELD			HAZ			BM		
	1	2	3	4	5	6	7	8	9	10	11	12	13	14	15
A	450	452	448	358	446	404	333	341	334	438	452	339	447	446	449
B	439	440	444	350	375	448	336	339	330	439	341	369	438	439	443

**Table 5:** Static tensile test results.

No.	Determination of Sample	Tensile Strength R_m_, MPa	Comments
1	R1	1229	rupture in HAZ
2	R2	1286	

**Table 6:** Chemical composition of particles.

Spot	C	O	Al	Ca	Fe	Si	Balance
1	11.37	38.07	18.38	15.15	13.70	2.63	0.70
2	11.81	38.41	17.57	15.63	12.76	2.99	0.83

**Table 7:** The results of the bending test on the S1300QL steel joints.

No.	Designation of Sample	Angle of Bend, °	Comments
1	FBB/I	35	Brittle cracking in HAZ
2	FBB/II	70	
3	RBB/I	65	
4	RBB/II	70	

FBB—face bending test, RBB—root bending test.

**Table 8:** Results of impact testing at −40 °C.

No.	Designation	Notch Type	Breaking Work, J	Impact Strength J/cm^2^	Fracture Characteristics
1	VWT/I	Charpy V	17	42.5	Mixed fracture
2	VWT/II		18	45.0	
3	VWT/III		15	37.5	
4	VHT/I		18	45.0	
5	VHT/II		24	60.0	
6	VHT/III		20	50.0	

VWT—impact strength in the weld, VHT—impact strength in the HAZ.

## Data Availability

Not applicable.
